# A comparison of performance enhancing synergy among ultrafiltered yeast extracts and recombinant human serum albumin in CHO-K1 cells

**DOI:** 10.1186/1753-6561-5-S8-P26

**Published:** 2011-11-22

**Authors:** James F Babcock, Karen A Benedict, Amanda L Perlman

**Affiliations:** 1 Sheffield Center for Cell Culture Technology, Sheffield Bio-Science, A Kerry Group Business, Ithaca, NY USA

## Introduction

We have previously demonstrated a synergistic reaction between a wheat hydrolysate and recombinant human serum albumin used to supplement a chemically defined growth medium for SP2/0 hybridoma cells. The data presented here illustrate the synergystic performance enhancing effect obtained when ultrafiltered yeast extract and recombinant human serum albumin are co-supplemented in CHO cell media. Each combination has its own distinctive effect on the growth and productivity of transfected cells. Cell viability, cell proliferation and target protein production all may be improved, yet these effects are not necessarily observed concurrently in a given system.

## Materials and methods

Data were collected using a transfected CHO-K1 line, adapted to serum-free suspension culture, and engineered to constitutively express secreted embryonic alkaline phosphatase (SEAP) by means of a modified human cytomegalovirus (hCMV) promoter.

Cultures were grown in 125 ml shake-flasks containing a final medium volume of 35 ml. The basal medium consisted of 100% chemically defined medium (CDM) supplemented with 1 mg/ml G-418. Triplicate flasks were seeded at 4.0 x10^5^ cells/ml, and incubated at 37°C in 5% CO_2_ at 130 rpm for 12 days. Medium supplement stock solutions were prepared at 100 g/l in the basal medium and sterilized through a 2.0 µm filter.

At days 5, 7, 8, 9 and 12, 1.0 ml of the culture supernatants were removed for assessing cell counts and viability. Cells were counted using a Nova BioProfile Flex automated analyzer. At Day 12, 1.0 ml of the culture supernatants were removed for SEAP analysis. Levels of SEAP in the supernatants were measured using anion-exchange HPLC.

## Results

**Figure 1:**
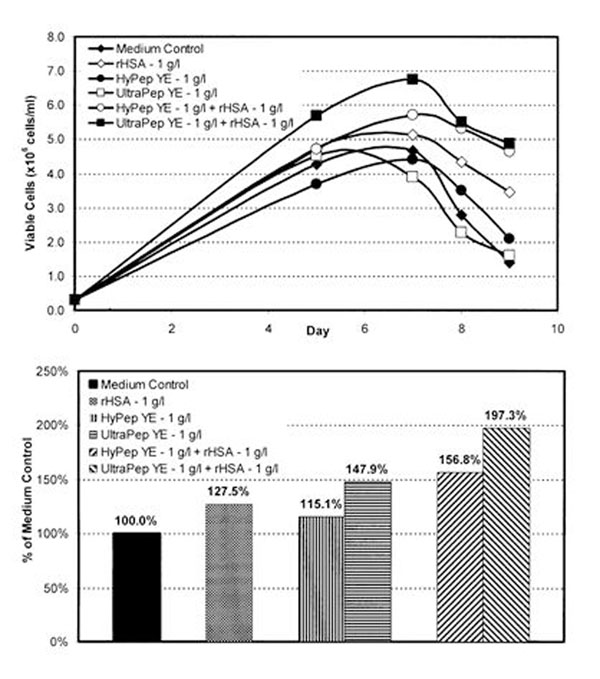
Growth Curves and SEAP Titers for rHSA, HyPep YE and UltraPep YE Supplemented Batch Cell Cultures

Maximum cell density increased with respect to the Medium Control when cultures were dosed with rHSA at 1 g/l, but not when supplemented with HyPep YE at 1 g/l. When both supplements were used together, an even greater increase in cell density was observed. The synergystic effect was also seen with rHSA and UltraPep YE. However, the UltraPep YE/rHSA combination out-performed the HyPep YE/rHSA with respect to maximum cell density (Figure 1). All cultures were assayed for total SEAP production on Day 12. When dosed at 1g/l, all of the supplements (HyPep YE, UltraPep YE and rHSA) yielded higher titers than the Medium Control. The greatest increases were seen when HyPep YE or UltraPep YE were used in conjunction with rHSA (Figure 1).

## Summary

The data presented here illustrate the performance-enhancing synergy that may be realized by supplementing various cell culture media with a combination of yeast extract and recombinant human serum albumin. When the two supplements are used together, cell culture performance results exceed those achieved when using each supplement individually. Overall performance was further improved by varying the individual dosages of yeast extract and recombinant human serum albumin. In four separate basal media, cell response to co-supplementation for each of the yeast extract/recombinant albumin combinations tested was shown to be both medium and dosage dependent. The optimized combination provided significant overall performance improvement in all media tested.

